# Internet-based interventions for adults with hearing loss, tinnitus and vestibular disorders: a protocol for a systematic review

**DOI:** 10.1186/s13643-018-0880-9

**Published:** 2018-11-23

**Authors:** Eldré W. Beukes, Vinaya Manchaiah, David M. Baguley, Peter M. Allen, Gerhard Andersson

**Affiliations:** 1 Department of Vision and Hearing Sciences, Anglia Ruskin University, Cambridge, CB1 1PT UK; 2 Department of Speech and Hearing Sciences, Lamar University, Beaumont, TX USA; 3 Department of Speech and Hearing, School of Allied Health Sciences, Manipal University, Manipal, Karnataka India; 4 Audiology India, Mysore, Karnataka India; 5 National Institute for Health Research, Nottingham Biomedical Research Centre, Ropewalk House, 113 The Ropewalk, Nottingham, UK; 6 Hearing Sciences, Division of Clinical Neuroscience, School of Medicine, University of Nottingham, Nottingham, UK; 7 Vision and Eye Research Unit, Anglia Ruskin University, Cambridge, UK; 8 Department of Behavioural Sciences and Learning, Linköping University, Linköping, Sweden; 9 Department of Clinical Neuroscience, Division of Psychiatry, Karolinska Institute, Stockholm, Sweden

**Keywords:** Internet interventions, eHealth, Self-help, Hearing loss, Vestibular disorders, Tinnitus, Systematic review, Protocol

## Abstract

**Background:**

Internet-based interventions are emerging as an alternative way of delivering accessible healthcare for various conditions including hearing and balance disorders. A comprehensive review regarding the evidence-base of Internet-based interventions for auditory-related conditions is required to determine the existing evidence of their efficacy and effectiveness. The objective of the current protocol is to provide the methodology for a systematic review regarding the effects of Internet-based interventions for adults with hearing loss, tinnitus and vestibular disorders.

**Method:**

This protocol was developed according to the Preferred Reporting Items for Systematic reviews and Meta-analyses for Protocols (PRISMA-P) 2015 guidelines. Electronic database searches will include EBSCOhost, PubMed and Cochrane Central Register performed by two researchers. This will be complemented by searching other resources such as the reference lists for included studies to identify studies meeting the eligibility for inclusion with regard to study designs, participants, interventions, comparators and outcomes. The Cochrane risk of bias tool (RoB 2) for randomised trials will be used for the bias assessments in the included studies. Criteria for conducting meta-analyses were defined.

**Discussion:**

The result of this systematic review will be of value to establish the effects of Internet-based interventions for hearing loss, tinnitus and vestibular disorders. This will be of importance to guide future planning of auditory intervention research and clinical services by healthcare providers, researchers, consumers and stakeholders.

**Systematic review registration:**

PROSPERO CRD42018094801

**Electronic supplementary material:**

The online version of this article (10.1186/s13643-018-0880-9) contains supplementary material, which is available to authorized users.

## Background

Chronic auditory conditions can be debilitating and greatly reduce quality of life [1]. They generally fall into three broad categories, namely hearing disability, tinnitus and vestibular disorders. The impact of hearing loss is often multifactorial and not isolated to reduced hearing and increased listening effort. For example, it can negatively impact on the ability to communicate, which amplifies social isolation, and can lead to relationship difficulties and reduced well-being [2, 3]. In addition, the presence of uncorrected hearing loss increases the risk of cognitive decline and dementia [4, 5]. For those with troublesome tinnitus, many aspects of daily life may be disrupted, leading to sleep and concentration difficulties, and indirect psychosocial effects, including feelings of hopelessness, irritability, frustration, anxiety and depression [6, 7]. Loss of vestibular function can cause imbalance, dizziness and an increased risk of falls [8]. This can affect the ability to carry out activities of daily living such as walking and driving. There is an increased dependence on others and decreased life satisfaction [9].

Auditory-related conditions are prevalent with around 15% of the world’s population having some degree of hearing loss [10]. In addition, hearing loss of greater than 20 dB was found to be the second most common impairment, from a systematic review investigating 310 diseases [11]. At least 10% of the adult population have tinnitus, as seen from studies across the globe, for example from Italy [12], Korea [13], New Zealand [14], the UK [15, 16] and the USA [17, 18]. The prevalence of dizziness has been reported to be 20–30% among adults [9, 19]. Although hearing loss, tinnitus and vestibular disorders can occur in isolation, they often co-occur and are associated with otological pathologies such as otosclerosis, Ménière’s disease, cerebellopontine angle lesions (such as vestibular schwannoma) and superior semicircular canal dehiscence [20]. In addition, the prevalence of auditory-related conditions generally increases with age [21–23]. This is a concern as the proportion of elderly people is rising [24]. These disabilities add to the healthcare and societal economic burden. Unaddressed hearing loss poses an annual global cost of $750 billion dollars [25]. The annual cost of tinnitus interventions in the UK was calculated to be £750 million in total and the annual societal costs relating to tinnitus was calculated at £2.7 billion [26]. In the USA, the annual economic burdens of unilateral and bilateral vestibular disorders were found to be $3531–$13,019 per patient [27].

As these are chronic long-term conditions, ongoing management over a period of years or decades is often required [25]. Interventions to prevent, identify and address hearing loss, tinnitus and vestibular disorders can be cost-effective [28–30] and can bring great benefit to individuals in reducing the adverse impact these difficulties have [31–33]. The standard intervention for hearing loss involves the provision of hearing aids within an audiology clinic [34]. Although hearing aids can help reduce the negative consequences of hearing loss, the uptake and adherence are suboptimal, even in countries where the provision of hearing aids is free at the point of use [35]. Despite moderate-to-strong evidence that vestibular rehabilitation is an effective treatment for peripheral vestibular disease [36], less than 3% of eligible primary care patients with dizziness ever received vestibular rehabilitation [37]. Moreover, the structure, provision of, and access to tinnitus services vary greatly depending on demographic location [38].

In an attempt to increase access to treatments and improve outcomes of rehabilitation for auditory-related disabilities, Internet-delivered interventions have been developed [39–42]. These interventions have generally focused on providing self-help techniques for behavioural change by means of a structured programme. Within the field of audiology, they have been developed to improve hearing aid use and/or reduce hearing disability [43]; reduce tinnitus distress through techniques such as cognitive behavioural therapy [44]; or improve balance function through vestibular rehabilitation [45]. These programmes generally last 6–10 weeks and may be independent of professional support (unguided) or offer some form of support (guided). The intervention content usually consists of a range of modules (6–21 chapters) with interactive elements such as quizzes and worksheets. Internet interventions for auditory-related conditions have a relatively short history with the first trials conducted in the field of tinnitus [42]. As such, evidence of their efficacy and effectiveness is still being sought. In 2010, Swanepoel et al. [46] conducted a broad-spectrum systematic review to identify telehealth applications for screening, diagnosis and interventions in audiology. In this review [46], the use of Internet-based interventions was only identified for tinnitus and not for hearing loss and vestibular disorders. Since then, additional studies related to Internet-based interventions in the fields of tinnitus have been published as well as in the fields of hearing loss and vestibular disorders. With this emergence of new evidence, an updated review is warranted. Within the hearing domain, there has been a systematic review investigating the efficacy of computer-based auditory training [46], but not Internet-based training. Reviews in the field of tinnitus have investigated the efficacy of cognitive behavioural therapy (CBT) for tinnitus [47], tinnitus management [48] or self-help tinnitus interventions [49]. No recent review has focused solely on Internet interventions for tinnitus. Moreover, systematic reviews for vestibular rehabilitation were found [50], but these were not specific to Internet-based interventions. Reviews that have investigated Internet interventions have either explored a specific health conditions (e.g. anxiety) or a variety of general health problems [51]. In view of the lack of an up to date and comprehensive review of the role of Internet interventions for auditory conditions, the current review protocol was designed. The aim of this protocol is to investigate the effects of Internet-based interventions for adults with hearing loss, tinnitus and vestibular disorders. This will include determining the efficacy of Internet-based interventions, referring to the extent to which an intervention produces a beneficial result under ideal conditions, as well as their effectiveness, which is the extent to which a specific intervention, when used under ordinary circumstances, does what it is intended to do [52]. The main research question was formulated following consultation with researchers in the field: what is the effect of Internet-based interventions for adults with hearing loss, tinnitus and vestibular disorder? Taking this broad-spectrum approach has disadvantages such as mixing different disorders which may have different intervention effects [53]. This approach is required due to the more recent nature of audiological Internet interventions and to provide a more comprehensive overview of these interventions and to identify whether further reviews with a narrower scope are indicated.

### Objectives

A systematic review related to Internet interventions for chronic auditory conditions of hearing loss, tinnitus or vestibular disorders will be undertaken to answer the following questions:

(i) What are the effects of Internet-based interventions post-intervention to reduce hearing disability, tinnitus distress and vestibular disorders in adults?
(ii) What are the effects of Internet-based interventions for adults post-intervention on associated difficulties of anxiety, depression, insomnia, and quality of life, often related to having a hearing loss, tinnitus, or vestibular disorders?
(iii) What are the effects of Internet-based interventions post-intervention to reduce hearing disability, tinnitus distress and vestibular disorders in adults one year after undertaking the intervention?

## Methods

This systematic review was prospectively registered with the International Prospective Register of Systematic Reviews (PROSPERO number CRD42018094801). The methods selected were guided by the Preferred Reporting Items for Systematic reviews and Meta-analyses for Protocols (PRISMA-P) [54, 55] (see Additional file 1). In the event of differences between the protocol and the completed review, these amendments will be presented together with the date of amendment, description of the change, rationale and consequence of these modifications.

### Eligibility criteria

Table 1 lists the study eligibility criteria. Consistent with the PRISMA-P statement, the inclusion criteria has been selected with reference to Participants, Interventions, Comparators, Outcomes, Timing and Study designs (PICOTS) [56] as well as criteria for the publication language and setting.

**Table 1:** Review eligibility criteria

Study characteristic	Inclusion criteria	Exclusion criteria
Study designs	• Randomised controlled trials (RCT) (both efficacy and effectiveness trials) • Crossover designs where data from before the cross-over are extractable to avoid the potential for a carry-over phenomenon	• Cluster randomised RCTs • Non-randomised trials • Repeated measures designs (pre- and post-intervention) unless this is for the long term outcomes after group cross- over has taken place or control conditions are no longer available • Quasi-experimental controlled trials • Case studies • Observational studies • Purely qualitative studies • Expert options • Cross-sectional studies • Trials were participants have not been randomly assigned
Participants	All adults (aged ≥ 18 years) from both clinical and non-clinical samples (self-referred due to response from study advertisement) with acute or chronic complaints of hearing loss, tinnitus and/or vestibular disorders and meeting the Intervention studies’ eligibility criteria. Adults with significant levels of disability as defined by the individual studies’ inclusion criteria to include: - A significant global score on a multi-item questionnaire (Table 3) - Presenting with hearing loss of at least a mild degree as measured by an audiologist using pure tone audiometric testing -Significant levels of hearing loss, tinnitus and/or dizziness diagnosed by an Ear Nose and Throat consultant, audiologist or clinical psychologist following clinical examination This assumes that those with significant co-existing conditions and undertaking co-interventions (excluding hearing aid fittings) will be excluded. All ethnic and social-economic groups will be included	• Data focused on children and adolescents • Studies not defining the eligibility criteria to undertake the Internet interventions for hearing handicap, tinnitus distress and vestibular difficulties, such as tinnitus of at least 3 months duration of moderate severity as measured by a self-reported assessment measure
Interventions	• Internet-based interventions as a structured form of self-help aimed at reducing difficulties related to hearing loss, vestibular disorders, and tinnitus • Both guided and self-guided interventions will be included • An element of blending may be involved such as introducing the intervention during a face-to-face consultation. However, the Internet-intervention part needs to be 70% or greater than the face-to-face part • There are no limitations based on the starting point of interventions or their durations • There should be a minimum of at least one Internet-intervention • Internet interventions running concurrently with hearing aid fittings will be included as this forms part of standard audiological care	• Predominantly app-based interventions • Solely computer-based programmes not accessed via the Internet (e.g. provided on disks/DVDs) • Interventions using a predominantly blended approach with 30% or more face-to-face input • Online discussion forums provided in isolation and not as part of a structured programme • Internet interventions running concurrently with additional treatments (excluding hearing aid fittings) will be excluded as the effects of the Internet intervention will not be isolated.
Comparators	At least one comparator is required this may be either an inactive control (e.g. no treatment, standard care, waiting list control, discussion forum, information only, usual care) or active control (e.g. different variant of the same intervention, a different kind of therapy)	• No comparison (single group designs) unless this is for the long term outcomes after group cross-over has taken place or control conditions are no longer available • Comparators comparing the role of guidance using the same Internet-based intervention in both the experimental and the control groups
Outcomes	Reporting results from a self-reported outcome measure related to the main difficulty targeted e.g. hearing loss, tinnitus, or vestibular difficulties	• Primary outcome reported not related to hearing loss, tinnitus, or vestibular difficulties • Primary outcome, not a self-reported measure
Timings	At least two data points are required for pre and post-intervention or follow-up (e.g. baseline and 1 year post-intervention) endpoint outcomes	No post-intervention follow-up period
Additional inclusion criteria		
Language	English only	
Setting	All settings including clinics, hospitals (private, public, university) and/or home-treatments in all geographic locations	

### Information sources

#### Electronic databases

A systematic search strategy will be used to search the following electronic research databases with no date restrictions for manuscripts published or accepted for publication in peer-reviewed academic journals:

- EBSCOhost including Allied and Complementary Medicine (AMED), Cumulative Index to Nursing and Allied Health Literature (CINAHL)
- PubMed (including MEDLINE)
- Cochrane Central Register of Controlled Trials database
- Embase

#### Other resources

Manual searches will be implemented to increase the comprehensive coverage of the available literate to ensure that all potentially eligible records will be identified. This will include:

- Trial registers and trial result registers at clinical.gov and Cochrane Ear, Nose and Throat Disorders Group Trials Register for completed trials that may be accepted for publication
- Hand-searching key journals and the reference lists from the included studies
- Grey literature will be searched in Google Scholar
- Contacting stakeholders such as researchers and experts in the field if any further records were outstanding or they have any manuscripts that have been accepted for publication

### Search strategy

The search strategy was developed together with an information specialist at Anglia Ruskin University to improve search quality [57, 58] and peer reviewed. A search strategy using medical subject headings (MeSH) terms to target four key domains—(i) condition (e.g. hearing loss, tinnitus and vestibular disorders); (ii) treatment (e.g. intervention, rehabilitation, self-help); (iii) mode of delivery (e.g. online, Internet-based, web-based); and study designs (randomised)—was identified. The use of search terms and its Boolean combination was adapted for each search engine to suit its requirements. Table 2 provides the MEDLINE search strategy that will be used to search titles and abstracts. The final search strategies will be included in the completed review. The literature searches will be conducted independently by two researchers, namely the first author and a research assistant independent of the review for comparative purposes. A pilot search test was undertaken for the tinnitus category first to ensure that the search strategy was effective.

**Table 2:** Search strategy for PubMed (MEDLINE) database

Condition	Search strategy
For hearing loss	(hearing loss OR deafness OR hearing impairment OR deaf* OR hard of hearing OR hear*) AND (intervention OR treatment OR therapy OR program OR strategy OR self-help OR rehabilitation) AND (Internet* OR online* or web*)
For tinnitus	(tinnitus*) AND (intervention OR treatment OR therapy OR program OR strategy OR self-help OR rehabilitation) AND (Internet* OR online* or web*)
For vestibular disorders	(vestibular* OR dizziness* OR balance* OR Ménière* OR labyrinthitis OR neuritis OR benign paroxysmal positional vertigo OR BPPV OR endolymphatic hydrops) AND (intervention OR treatment OR therapy OR program OR strategy OR self-help OR rehabilitation) AND (Internet* OR online* or web*)
Limiters	English Language; Human Search modes: Boolean/ phrase

Limitations of this search strategy include the language restrictions and financial constraints preventing an expert to do the database searches.

### Study records

#### Data management

Identified records will be downloaded into a master file using RefWorks that will enable records to be tracked through the screening and data collection process and will remove duplicate records. Only exact duplicates will be removed. Multiple publications of the same study will be checked for relevance as different study characteristics may be reported in each publication. Included records will be allocated a study identification code to link each record with its corresponding full text and data collection sheet. The title and abstracts of the publications will be assessed against the inclusion criteria. Reasons for including or excluding publications will be documented and presented in a PRISMA flow diagram.

#### Selection process

Materials downloaded from electronic sources will include details of authors, journal and the abstract. Where in doubt, the full article will be sought. Articles appearing to meet the inclusion criteria will be retrieved to ensure they meet the inclusion criteria for this review. Two reviewers (EB and VM) will independently select articles for inclusion. Any disparities will be run by a third reviewer (GA). For any remaining disparities, the full team will discuss these to reach a conclusion (EB, VM, PA, DB, GA). A flow diagram will be used to summarise the studies included and excluded from the review. Excluded articles and the rationale for exclusion will be presented in the completed review. Study selection methods were conducted on a pilot group of studies to calibrate reviewers (EB and VM) and to fine-tune eligibility criteria.

### Data collection

Data from selected studies will be recorded on a data extraction form using the PICOTS format (Participants, Interventions, Comparators, Outcomes, Timing and Study design) [56, 59]. The form was piloted by EB and VM and verified by VM. Following piloting, the need to extract additional study characteristics (e.g. duration of disorder and mean pure tone average data) was identified. Data will be extracted by one reviewer (EB) and verified by another reviewer (VM). The complete extraction sheet will be provided to all other authors for cross-checking.

### Data items

The Cochrane data collection form for intervention studies with a randomised controlled trial format was used during the development of the extraction forms. The forms were tailored for the research questions of this review. The data items that will be collected can be found in Additional file 2. If both intention-to-treat and per-protocol data were presented, the intention-to-treat estimation will be used.

### Outcomes and prioritisation

As assessing disability associated with auditory conditions is generally through use of patient-reported outcome measures (PROM), these questionnaire results will be used in assessing the outcome regarding each intervention type. For each outcome measure, there is more than one possible method of assessment. Those measuring similar domains have been selected for this review which will allow for later data synthesis if there are sufficient studies with comparable data. The PROMs used in each study will be documented. The effects of Internet interventions will be assessed in terms of the following outcomes, in order of prioritisation, as identified following consultation with experts and researchers in the field. Otological conditions and related health problems often co-occur and can be regarded as composite health problems [60]. As the interventions for this review are focused specifically on either hearing difficulties, tinnitus or vestibular difficulties, it is unlikely that they will include composite outcomes. This review will focus only on the following primary and secondary outcomes:

#### Primary outcomes

The effects of Internet-based interventions will be assessed by comparing the mean difference at post-intervention (immediately after the intervention has been completed) between scores for the experimental and control groups for hearing disability, tinnitus distress or dizziness as indicated by a PROM detailed in Table 3.

**Table 3:** Examples of questionnaires measuring primary outcomes for this review. This list will be updated if other questionnaires are introduced

Measurement instrument (author, year)	Number of items and subscales	Internal consistency (Cronbach’s alpha for the global score)
Hearing handicap		
Hearing Handicap Inventory for the Elderly [85]	25 items, 2 subscales	*a* = 0.93
Hearing Handicap Questionnaire [86]	27 item, 3 subscales	*a* = 0.94
Tinnitus distress/severity		
Tinnitus Handicap Inventory [87]	25 items, 3 subscales	*a* = 0.93
Tinnitus Questionnaire [88]	52 items, 5 subscales	*a* = 0.94
Tinnitus Reaction Questionnaire [89]	26 items, 4 subscales	*a* = 0.96
Tinnitus Functional Index [90]	25 items, 8 subscales	*a* = 0.97
Tinnitus Handicap Questionnaire [91]	27 items, 3 subscales	*a* = 0.94
Vertigo/dizziness		
Vertigo Symptom Scale-Short Form [92]	36 items, 2 subscales	*a* = 0.90
Vestibular Rehabilitation Benefit Questionnaire [93]	36 item, 4 subscales	*a* = 0.73
Dizziness Handicap Inventory [94]	25 items, 3 subscales	*a* = 0.89
Vertigo Handicap Questionnaire [95]	25 items, 4 subscales	*a* = 0.93

#### Secondary outcomes

The effects of intervention-based interventions by comparing the mean difference immediately after the intervention has been completed (post-intervention) between scores for the experimental and control groups for difficulties often related to having a hearing loss, tinnitus or vestibular disorders namely:

- Anxiety as measured by a validated instrument such as the anxiety scale of the Hospital Anxiety and Depression Scale (HADS) [61] or the Depression, Anxiety and Stress Scales (DASS) [62], the Generalised Anxiety Disorder [63] or the Beck Anxiety Inventory [64]
- Depressive symptoms or depression as measured by a validated instrument such as the depression scale of the HADS [61] or the DASS [62], Patient Health Questionnaire [65] or the Beck Depression Inventory [66]
- Insomnia as measured by a validated instrument such as the Insomnia Severity Index [67]
- Quality of life as measured by a validated instrument such as the Satisfaction With Life Scales [68], Quality of life Inventory [69] or The World Health Organization Quality of Life assessment [70]

#### Long-term outcomes

To determine the long-term outcomes, 1 year or longer post-intervention for hearing disability, tinnitus distress and dizziness using a PROM from Table 3. This is likely to be comparing the mean difference scores between pre-intervention and 1 or more year’s follow-up as crossover designs may have been used where the control groups would have had treatment by this point. Long-term outcomes will be divided into subgroups according to the time points for measuring these outcomes, e.g. 12 months, 18 months, 24 months post-intervention.

### Risk of bias in the individual studies

The risk of bias for the included studies will be assessed using the Cochrane Collaboration’s tool (RoB 2) for randomised trials [71]. Included studies will be assessed for bias across the following five domains: (1) bias arising from the randomisation process; (2) bias due to deviations from intended interventions; (3) bias due to missing outcome data; (4) bias in measurement of the outcome; (5) bias in selection of the reported result. Each item will be judged as yes, probably yes, probably no, no and no information by two reviewers (EB and VM). Any discrepancies will be resolved by discussion and then by consulting with a third reviewer (GA). An overall risk of bias judgement will be made as low risk of bias, some concerns or a high risk of bias.

### Data synthesis

The criteria for conducting a quantitative synthesis will include:

1. Each included study addresses the same question
2. A low risk of bias in the included studies
3. Consistent outcomes between studies
4. Low publication bias
5. A high number of included studies and
6. Low heterogeneity

These criteria will be collectively analysed when deciding to undertake a meta-analyses or not. We will apply the random effects model, as study heterogeneity is expected. We will explain the rationale for a possible change from the random effects to the fixed effect model in the completed review. Comprehensive Meta-Analysis software version 3 [72] will be used to conduct the meta-analyses.

#### Summary measures

Studies with more than one active treatment arm will be aggregated and analysed separately. The characteristics of the included studies will be summarised according to the characteristics of the study designs, participants, interventions, comparators and outcomes and timings. The standardised mean difference (Cohen’s *d* effect size) will be used when different scales of measurements have been used to measure the same outcome. A positive effect size will indicate that the Internet-intervention group achieved better outcomes than the control group. A forest plot will be constructed to visualise the effect sizes, confidence intervals and heterogeneous nature of the included studies where 10 or more studies are included [73].

#### Unit of analysis issues

Unit of analysis issues could arise due to (1) the level of randomisation, (2) use of multiple observations and (3) trials with multiple groups. To address the first unit of analysis issue, the primary analyses will be per randomised individual and cluster-randomised trials will be excluded. To address multiple observations, a single time point at immediately post-intervention has been selected for the secondary outcomes to avoid this issue. For the primary outcomes, a single time point at immediately post-intervention is selected and the longest follow-up, only if this is at least 1 year post-intervention to reduce analysis issues. For trials with complex data structures such as multiple independent subgroups within a study, multiple outcomes or time-points within a study, or more than one comparison group within a study, we will consult the various statistical approaches described by Borenstein et al. (2009a, 2009b, 2009c, 2009d), Higgins et al. (2011) and Shuster (2011) [74–79] and guidance from a statistician will be sought and a rationale will be presented for the methods implemented.

#### Missing data

Where data is missing or unclear from the published studies, an effort will be made to obtain this information from the trial authors to a maximum of three attempts. When authors do not reply or are unable to provide us with this information, we will assess whether data were missing at random or not. Sensitivity analyses will be performed to assess the potential impact of missing data and how best to address these missing data [79].

#### Clinical heterogeneity

The psychometric properties of the outcome measures used will be considered with regard to their suitability for pooling. Data will only be pooled if the assessment measures have the same underlying constructs regarding participants, interventions, comparators, outcome measurements, timing, setting etc. If appropriate, the mean difference with 95% CI will summarise the pooled analyses for the included studies using the mean between-group post-intervention scores (or mean change from baseline to follow-up for 1 year + outcomes) and standard deviations [80].

#### Statistical heterogeneity

Consistency between studies will be explored using the *Q* value and *I*^*2*^ statistic values. The *I*^2^ statistic results will be broadly categorised as suggested by Higgins [81] on a range of 0–100% (25% low, 50% moderate and 75% high). A *p* value < 0.1 will be considered statistically significant. If substantial heterogeneity is identified, this will be explored through the pre-specified subgroup analyses and sensitivity analyses, where sufficient data permits. Tau^2^ will be used to measure variance.

#### Additional analyses

If sufficient data are available, subgroup analyses will be performed for the categorical variables:

- *Study designs:* effectiveness and efficacy, separating those with inactive and active comparators.
- *Participants*
  Age: young adult, adults, the elderly.
  Populations: veteran versus non-veteran
- *Intervention type:* hearing loss, tinnitus, vestibular
- *Outcomes:* primary and secondary (anxiety, depression, insomnia, quality of life) at post-intervention and long-term outcomes for the primary outcomes (≥ 1 year outcomes)

A sensitivity analyses will be conducted by excluding those studies with a high risk of bias, thereby determining the robustness of the conclusions from the included studies. Assessing how outcomes of studies from specific (collaborating) research groups influence the summary effect size is also planned.

#### Meta-regression

Meta-regression will be used to investigate statistical heterogeneity. Meta-regressions will be conducted to examine the impact of different study characteristics on the study effect size. Meta-regressions will be considered where there are ten or more studies.

#### Qualitative (narrative synthesis)

If a quantitative synthesis is not appropriate, a systematic narrative synthesis will be provided to explain the characteristics and findings of the included studies using text and tables to aid conceptual understanding of the data for each research question. The narrative synthesis will explore the relationship and findings both within and between the included studies.

### Meta-biases

The following strategies for assessing and dealing with selective outcome reporting will be applied:

1. The protocols of eligible studies will be assessed
2. Differences between protocols and the final study will be identified
3. Authors will be contacted to obtain additional information where required
4. Missing data will be analysed to determine whether it is missing at random or not. This will determine the most appropriate way of dealing with the missing data [74].

Publication bias will be explored using funnel plots. Asymmetry in the funnel plots will only be assessed when ten or more eligible studies are identified, because with fewer articles, the power of this statistic is too low. Orwin’s fail-safe N procedure will be used to numerically identify bias. Duval and Tweedie’s trim and fill iterative procedure will be used to remove the most extreme studies from the positive side of the funnel plot and re-compute the effect size [82].

### Confidence in the cumulative estimate

Judgements about the quality of the evidence for each research question will be rated according to the Grading of Recommendations Assessment, Development and Evaluation (GRADE) protocol [83]. The level of evidence will be scored to be either high quality, moderate quality, low quality or very low quality. These judgements will be made independently by two reviewers (EB, VM). The lower the score the less confidence in the effect estimate, the higher the score the more confidence can be applied that the true effect lies close to that of the estimate of the effect.

## Discussion

The limited availability, accessibility and affordability of hearing healthcare have recently been highlighted [84]. Applications of technological advances have been incorporated as a way of improving healthcare. Internet interventions are one such example that have been used recently for auditory-related conditions. In view of recent developments, assessing the evidence-based supporting an Internet-based intervention format is important. This planned review is thus of value to establish the current effects of Internet interventions within audiology. This information is required to assist the future planning of accessible evidence-based audiological healthcare services. This review will thus be of value to stakeholders and clinical services and help guide further research. It is also important to help consumers of these interventions to know their possible effects. The previous review conducted in 2010 [46] included all telehealth application within the scope of audiology from screening through to diagnosis and treatment. This present review will focus only on Internet-based interventions within audiological telehealth applications. Although this scope is more focused, it is still a broad-spectrum approach by including Internet-based interventions for hearing loss, tinnitus and vestibular disorders. Mixing different disorders which could have different intervention effects is a limitation but was selected as audiological Internet interventions do not have a long history. There is therefore the possibility that there will be too few studies available to draw valid conclusions from if the focus is on individual disorders. If enough studies are found further suggestions for follow-up reviews with a narrow scope will be made. Due to the relative newness of audiological Internet interventions, treatment credibility may not yet be established from both patients’ and clinicians’ viewpoints. This review may aid knowledge regarding the effects of these interventions. There is also the danger that optimal sample sizes have not being recruited as treatment credibility may not yet be established. Hence, the review will include the reporting of low powered studies. The drop-out rates in the included studies may also be high, introducing further bias. Moreover, the possibility that the interventions will have been developed and conducted by the same research groups and this potential source of bias will be considered. This review will be limited to English due to time and financial constraints. This may introduce the risk of publication bias, and the results need to be interpreted with this consideration. Limitations of this search strategy include the language restrictions and financial constraints preventing an expert to do the database searches. Despite these limitations, this review is important for the future planning of accessible, affordable and evidence-based interventions for distressing symptoms related to having hearing loss, tinnitus or vestibular disorders.

## Additional files

Additional file 1: PRISMA-P 2015 Checklist for reporting of systematic reviews (DOCX 29 kb)

Additional file 2: Data items that will be collected (DOCX 33 kb)

## Abbreviations

AMED: Allied and Complementary Medicine
CINAHL: Cumulative Index to Nursing and Allied Health Literature
PRISMA-P: Preferred Reporting Items for Systematic reviews and Meta-analyses for Protocols
PROM: Patient-reported outcome measures
PROSPERO: Prospective Register of Systematic Reviews
RCT: Randomised controlled trial
