# Supplementary material for: Internet-based interventions for adults with hearing loss, tinnitus and vestibular disorders: a protocol for a systematic review
Source: Syst Rev. 2018 Nov 23;7:205. doi: 10.1186/s13643-018-0880-9 (PMC6260838; doi:10.1186/s13643-018-0880-9)
Supplement: Supplementary file 2 — Data items that will be collected (DOCX 33 kb) [file 13643_2018_880_MOESM2_ESM.docx]

# Additional file 1

# Title: Internet-based interventions for adults with hearing loss, tinnitus and vestibular disorders

# Trial registration no: PROSPERO CRD42018094801

# Data collection forms

1. **General information**

| **Category** | **Description** |
| --- | --- |
| Study reference | List authors, title, and journal, publication date |
| Study identification number |  |
| Date review completed |  |
| Completed by |  |
| Reviewed by |  |
| Study author contact details |  |
| Study author contacted | Dates and nature of contact |
| Sources of funding |  |
| Possible conflicts of interest | For study authors |

**2. Eligibility**

| **Category** | **Description** | **Location in Text** |
| --- | --- | --- |
| Target condition | Hearing loss, tinnitus or vestibular disorders |  |
| Stage of treatment pathway | Pre-intervention, new hearing aid users, experiences hearing aid users |  |
| Study design and type | Efficacy or effectiveness study |  |
| Treatment arms | Number and type of treatment arms |  |
| Setting and population sample | Clinic, self-referred |  |
| Key conclusions | Main study findings |  |
| Eligibility | Confirm eligibility or reasons for exclusion |  |

**3. Participants**

| **Entry** | **Description and character of the information** | **Location in Text** |
| --- | --- | --- |
| Sample size calculations provided | Calculations for sample size determination |  |
| Number of participants | Number randomised and present in each group |  |
| Mean age | Mean age in years and months, standard deviations and ranges for participants and ratio in each subgroup |  |
| Gender | Present the genders represented and ratio in each subgroup |  |
| Duration of disorder | Present in years, months together with standard deviations and ranges |  |
| Country and location | Country and the city where the study was conducted |  |
| Study inclusion criteria | Study inclusion criteria |  |
| Study exclusion criteria | Study exclusion criteria |  |
| Exclusions | Participants excluded |  |
| Methods of recruitment | How was recruitment approached |  |
| Baseline imbalances | Any baseline differences that need to be accounted for |  |
| Withdrawals | Number of participants form each group withdrawing or not completing outcome measures |  |

**4.Interventions**

| **Entry** | **Description and character of the information** |
| --- | --- |
| Intervention type | Type of intervention |
| Theoretical basis | theoretical basis for the intervention content |
| Treatment duration | Treatment dose and frequency |
| Provision of guidance | Profession, number of professionals |
| Intervention delivery mode | Whether a blended approach was included and the dose and frequency thereof |
| Comparator | Type of comparator |
| Comparator duration | Dose and frequency of comparator |
| Co-interventions | Hearing aid fittings and other interventions |

**5. Outcomes**

| **Entry** | **Description and character of the information** | **Located in the text** |
| --- | --- | --- |
| Primary outcome | What is the primary outcome being assessed |  |
| Primary outcome measure | Name of the measure/s selected |  |
| Time points measure | Time points of measuring  primary outcome intervals such as 3, 6, 12 months post-intervention |  |
| Secondary outcome | What are the secondary outcome/s being assessed |  |
| Secondary outcome | Name of the secondary outcome measure/s selected for depression, anxiety, insomnia and quality of life used |  |
| Handling missing data | ITT analysis used and type |  |
| Adverse effects | Record reporting of adverse events |  |

**6. Results: Experimental intervention and comparator**

|  | **Experimental intervention (for each active Internet-intervention)** | | | | **Comparator** | | | |
| --- | --- | --- | --- | --- | --- | --- | --- | --- |
| **Entry** | **Mean** | **SD** | **Effect size** | **Number of participants** | **Mean** | **SD** | **Effect size** | **Number of participants** |
| Primary outcome: Baseline |  |  |  |  |  |  |  |  |
| Primary outcome: Post-intervention |  |  |  |  |  |  |  |  |
| Primary outcome: latest- follow-up |  |  |  |  |  |  |  |  |
| Depression outcome: baseline |  |  |  |  |  |  |  |  |
| Depression outcome: post-intervention |  |  |  |  |  |  |  |  |
| Anxiety outcome: baseline |  |  |  |  |  |  |  |  |
| Anxiety outcome: post-intervention |  |  |  |  |  |  |  |  |
| Insomnia outcome: baseline |  |  |  |  |  |  |  |  |
| Insomnia outcome: post-intervention |  |  |  |  |  |  |  |  |
| QOL outcome: baseline |  |  |  |  |  |  |  |  |
| QOL outcome: post-intervention |  |  |  |  |  |  |  |  |

**7. Risk of bias assessment**

| Domain | Risk of bias | | | Support for judgement  *(include direct quotes where available with explanatory comments)* | Location in text |
| --- | --- | --- | --- | --- | --- |
|  | Yes | Probably yes | Probably No NoInfo  no |  |  |
| Random sequence generation |  |  |  |  |  |
| Allocation concealment |  |  |  |  |  |
| Deviations from intended interventions |  |  |  |  |  |
| Incomplete outcome data |  |  |  |  |  |
| Bias in the measurement of outcome |  |  |  |  |  |
| Selective outcome reporting? |  |  |  |  |  |
| Overall risk of bias judgment | Low risk | Some concerns | High risk of bias |  |  |
| Predicted direction of bias | Favours experimental | Favours comparator | Unpredictable | Towards the null | Away from the null |
